# mHealth Interventions for Self-management of Hypertension: Framework and Systematic Review on Engagement, Interactivity, and Tailoring

**DOI:** 10.2196/29415

**Published:** 2022-03-02

**Authors:** Weidan Cao, M Wesley Milks, Xiaofu Liu, Megan E Gregory, Daniel Addison, Ping Zhang, Lang Li

**Affiliations:** 1 Department of Biomedical Informatics The Ohio State University Columbus, OH United States; 2 Division of Cardiovascular Medicine The Ohio State University College of Medicine Columbus, OH United States; 3 The Center for the Advancement of Team Science, Analytics, and Systems Thinking (CATALYST) The Ohio State University College of Medicine Columbus, OH United States

**Keywords:** mHealth, mobile app, digital behavior change, interventions, systematic review, hypertension, engagement, interactivity, tailoring, mobile phone

## Abstract

**Background:**

Engagement is essential for the effectiveness of digital behavior change interventions. Existing systematic reviews examining hypertension self-management interventions via mobile apps have primarily focused on intervention efficacy and app usability. Engagement in the prevention or management of hypertension is largely unknown.

**Objective:**

This systematic review explores the definition and role of engagement in hypertension-focused mobile health (mHealth) interventions, as well as how determinants of engagement (ie, tailoring and interactivity) have been implemented.

**Methods:**

A systematic review of mobile app interventions for hypertension self-management targeting adults, published from 2013 to 2020, was conducted. A total of 21 studies were included in this systematic review.

**Results:**

The engagement was defined or operationalized as a microlevel concept, operationalized as interaction with the interventions (ie, frequency of engagement, time or duration of engagement with the program, and intensity of engagement). For all 3 studies that tested the relationship, increased engagement was associated with better biomedical outcomes (eg, blood pressure change). Interactivity was limited in digital behavior change interventions, as only 7 studies provided 2-way communication between users and a health care professional, and 9 studies provided 1-way communication in possible critical conditions; that is, when abnormal blood pressure values were recorded, users or health care professionals were notified. The tailoring of interventions varied at different aspects, from the tailoring of intervention content (including goals, patient education, advice and feedback from health professionals, reminders, and motivational messages) to the tailoring of intervention dose and communication mode. Tailoring was carried out in a number of ways, considering patient characteristics such as goals, preferences, disease characteristics (eg, hypertension stage and medication list), disease self-management experience levels, medication adherence rate, and values and beliefs.

**Conclusions:**

Available studies support the importance of engagement in intervention effectiveness as well as the essential roles of patient factors in tailoring, interactivity, and engagement. A patient-centered engagement framework for hypertension self-management using mHealth technology is proposed here, with the intent of facilitating intervention design and disease self-management using mHealth technology.

## Introduction

### Background

Hypertension is an impactful risk factor for heart disease and stroke, both of which are leading causes of death in the United States [1]. Approximately 45% of American adults have a diagnosis of hypertension, and only 24% of those with hypertension have their condition under optimal control [1]. Effective treatment of hypertension requires patients to work with their health care providers and follow self-management guidelines, particularly relating to medication adherence.

Mobile health (mHealth) is defined as the use of mobile technologies (eg, smartphones) to provide medical and public health practice [2]. mHealth interventions used for disease self-management belong to digital behavior change interventions, defined as those involving digital technologies (eg, mHealth apps) to promote or support behavior change for improved health and self-management of chronic disease [3,4] for better health, which have been used to facilitate hypertension self-management. Potential benefits of mHealth interventions for disease self-management include (1) increasing medication adherence [5], (2) increasing knowledge, (3) empowering patients for self-care, (4) providing personalized self-care recommendations, and (5) facilitating patient–care provider communication and decision-making [6,7]. Hundreds of mHealth apps, often with features such as educational resources and monitoring reminders, have previously been developed to support hypertension self-management [8], and studies have demonstrated the effectiveness of using apps in blood pressure (BP) control and self-management behavior change such as medication adherence [6,9,10].

Existing systematic reviews examining the mHealth interventions for hypertension self-management or digital behavior change interventions using mHealth in the context of hypertension have focused on intervention efficacy and app usability [6,9,11]. However, studies examining engagement in the context of hypertension are limited. Engagement can be defined from a microlevel perspective (ie, engagement with digital behavior change interventions only, such as intervention use, or the subjective experience characterized by attention, affect, and interest) or from a macrolevel perspective (ie, engagement with the broader landscape of behavior change, such as medication adherence) [3,4]. Macrolevel engagement can be the result of the microlevel of engagement. For instance, engagement (eg, frequency, amount, and duration) with interventions can lead to behavior engagement or change (eg, medication adherence). Engagement is essential for the effectiveness of interventions involving digital technology [3,12,13], whereas lack of engagement with mHealth interventions would be expected to be associated with a lower rate of intervention success [14]. Subsequently, understanding the determinants of engagement can help design effective interventions. Theoretical frameworks [15,16] have proposed specific strategies to engage patients using mobile apps. Such strategies include but are not limited to providing educational information, reminding or alerting users, recording and tracking health information, providing guidance based on information entered by the user, enabling communication with clinicians, providing support through social networks, and supporting behavior change through rewards [15,16].

Although prior work has examined engagement in the context of chronic conditions [17,18], it has largely focused only on the microlevel of engagement [17,18], thus leaving the macrolevel predominantly unexamined. Intervention effectiveness measured by app use alone cannot be taken as a valid indicator of engagement because use metrics (microlevel engagement) do not indicate offline engagement indicators, and microlevel disengagement with the intervention or technology does not necessarily preclude macrolevel engagement (eg, users may take medications adherently but do not use the app to track medication taking behaviors) [4].

Therefore, it is of vital importance to examine both types of engagement as well as their determinants in mHealth behavior change interventions [19]. According to the motivational technology model [20], customization (ie, tailoring) and interactivity are the two key determinants of engagement. Tailoring refers to the extent to which users can customize the mHealth intervention to meet their needs [21]. For instance, an app may tailor the educational content delivered, messages, alerts, and reminders, and displays to users’ specific needs and preferences; a patient on medication to manage severe hypertension likely requires different features and messaging than a patient who is managing mild hypertension through lifestyle modifications. Interactivity refers to the opportunities that the mHealth intervention affords for users to communicate with others, especially health care professionals [21]. For example, apps that have coaching from a trained professional tend to be more interactive. Along these lines, systematic reviews focusing on mHealth disease self-management interventions found that effective interventions integrated features of interactive communication [10] and tailored messages [22]. A systematic review of nutrition apps found that tailoring the apps to the needs of specific user groups can be beneficial in increasing engagement [23]. In addition, interventions can be more engaging if they are designed to be tailored to participants’ health beliefs and needs. For instance, a systematic review of studies on health beliefs and medication adherence in patients with hypertension found that medication adherence was related to health beliefs that vary within and across countries, such as disease severity and susceptibility, medication necessity, or efficacy [24]. This implies that medication adherence interventions need to consider individual health beliefs about hypertension and BP medications [24].

### Objective

Therefore, based on the importance of engagement, the research gap, the determinants of engagement, and the strategies to engage users, the following research questions were proposed:

1. What engagement strategies have been used in digital behavior change interventions for hypertension self-management?
2. How has engagement been defined or presented in the literature on digital behavior change interventions for hypertension self-management?
3. How has interactivity been implemented in digital behavior change interventions for hypertension self-management?
4. How has tailoring been implemented in digital behavior change interventions for hypertension self-management?

## Methods

### Overview

A systematic review of the literature was conducted to identify extant interventions and to investigate their key characteristics. Subsequently, content analyses of the studies were conducted for a deeper understanding of how engagement and its determinants were implemented in the interventions.

### Search Strategy

The search focused on the identification of studies relating to mHealth interventions for hypertension self-management conducted worldwide. The search was conducted on the following databases: PubMed, PsycINFO, Embase, Communication and Mass Media Complete, CINAHL, MEDLINE, and MEDLINE Full Text. All articles indexed as of June 2020 were searched. A combination of search terms was used, including *hypertension*, *hypertensive*, *hypertensives*, or *blood pressure* (for the disease type); *self-management*, *self management*, *self-care*, *self care*, *management*, *coaching*, *control*, *monitor*, *adhere*, or *adherence* (for disease management); *mHealth*, *m-health*, *mobile*, *app*, *apps*, *application*, *applications*, *smart phone*, *smartphone*, *technology* (for mHealth); *intervention*, *trial*, *program*, *programme*, *experiment*, *pilot*, *study*, *effect*, *experience*, or *experiences* (for intervention). Please refer to Multimedia Appendix 1 for the search strategy and the corresponding justifications.

To ensure the comprehensiveness of the search, we also scanned relevant journals (eg, JMIR mHealth and uHealth) and the reference lists of review articles about these interventions.

### Study Selection

Included studies were those conducted among adults (aged ≥18 years), involving a mobile app to facilitate hypertension self-management, and with the aim of testing app or system experience. If an app was designed specifically for hypertension management for patients with hypertension, then the study was included (eg, the study by Kang and Park [25]). If an app was used for BP reduction or hypertension and another health condition (eg, weight management), the study was included (eg, the study by Mao et al [26]). If an app was designed for a specific purpose (eg, medication adherence) and could be applied to different health conditions or diseases, and was applied to patients with hypertension in the study, the study was included (eg, the study by Morawski et al [27]). If an app was used for >1 disease (eg, for both diabetes and hypertension) and if patients with hypertension or patients with both conditions were included as participants in the study, then the study was included [14]. If an app was used among patients with other diseases or conditions (eg, kidney transplant) and managing hypertension is crucial for that disease or condition, then the study was included (eg, the study by McGillicuddy et al [28]).

The exclusion criteria included articles that met one or more of the following characteristics: (1) use of apps for the purposes of disease screening or disease detection; (2) focus only on app design and development, without reporting any participants’ app use experience; (3) primarily designed for healthcare providers or that reported professionals’ user experience but did not focus on patients’ user experience; (4) study of children; (5) based solely on non–smart phones, on the internet, or on text messages; (6) not written in English, and (7) contained only an abstract, without full publication. Covidence [29] was used to manage the review process.

**Figure 1:**
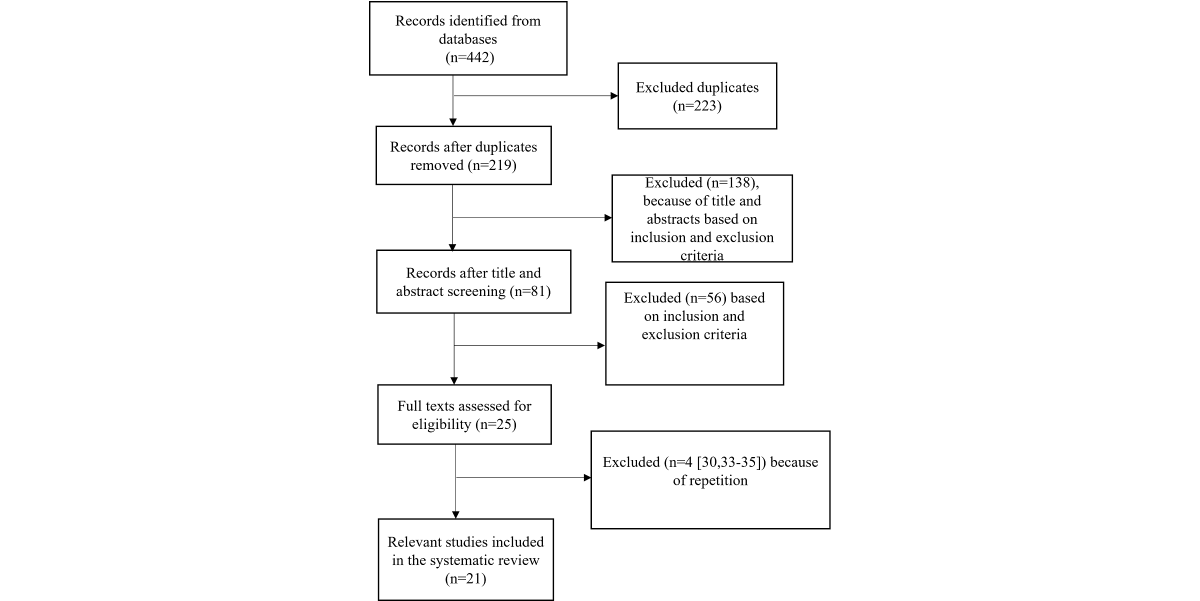
PRISMA (Preferred Reporting Items for Systematic Reviews and Meta-Analyses) flowchart.

A total of 442 records were imported to Covidence, and 223 duplicates were removed. After title and abstract screening, among the remaining 219 articles, 138 (63%) were removed based on not meeting the inclusion criteria. The remaining 37% (81/219) articles were screened, and 69% (56/81) of them not meeting the inclusion criteria were removed at this stage. A further review of the remaining 31% (25/81) of the articles indicated that 16% (4/25) of the articles [30-33] were based on the same app or intervention system. The study by Bengtsson et al [30] was not included in this review because it focused more on system development. The other study [33] was not included in this review because the patient participants were a subgroup of the patient participants in another study [32]. Furthermore, the studies by McGillicuddy et al [28,34] were based on the same system, and one of these studies [34] was not included because the other study [28] built on the 3-month randomized control trial conducted by McGillicuddy et al [34] and was a follow-up of that study. In addition, studies by Persell et al [35,36] were based on the same mobile app, so one of the studies [35] was removed because it focused more on the design of the app. Furthermore, the studies by Moore et al [37] and Thies et al [14] were based on the same app, and the studies by Chandler et al [38] and Davidson et al [39] were based on the same smartphone medication adherence stops hypertension program. For those studies that used the same system or app, if the participants, goals or outcomes, or methods (eg, surveys or interviews) were different, they were included in the review. For instance, although Moore et al [37] and Thies et al [14] used the same system, Moore et al [37] provided positive evidence that the system was effective in hypertension management, whereas Thies et al [14] analyzed the reasons why their intervention failed by using participant interviews. Including both articles would allow a full understanding of the effectiveness of the app or system. Therefore, the final sample size of the review is 21. A total of 2 authors (ie, WC and XL) worked independently during the screening and selection process first and then compared their results. Discrepancies were resolved through one round of discussion. Figure 1 presents the PRISMA (Preferred Reporting Items for Systematic Reviews and Meta-Analyses) flowchart.

### Quality Assessment and Data Analysis

For quality assessment, 2 coders (WC and XL) independently evaluated the quality of the included studies using four sets of risk of bias evaluation tools: Cochrane Collaboration’s Risk of Bias Tool for randomized control trials [40], 2 tools from the National Institutes of Health for observational studies and pre-post studies without a control group [41], and the critical appraisal skills program for qualitative studies [42]. For the study [43] that focused on both qualitative and quantitative data, we used two sets of criteria (ie, pre-post study with no control and qualitative study) to evaluate the risk of bias. These tools were chosen because they have been applied to previously published systematic reviews [9]. Disagreements between the coders were resolved after rounds of discussion and consultation with a third researcher (PZ). The results of the evaluation are in Multimedia Appendix 2 [27,36-39,44-47], Multimedia Appendix 3 [26,28,48,49], Multimedia Appendix 4 [25,31,43,50-52], and Multimedia Appendix 5 [14,32,43].

For the systematic review, 2 coders (WC and XL) independently coded the 21 studies. After independent coding, discrepancies were identified and resolved through multiple rounds of discussion and recoding. Through this iterative process, full agreement was reached for all variables of the systematic review. For coding, the investigators relied on the reporting in the article and referred to related articles listed in the references when applicable (eg, when coding an app that was published in multiple papers). If studies included both patients’ and providers’ perspectives, only the patients’ perspectives were coded. In the event that the article presented no relevant information or the description was general or vague, we coded it as *unknown*.

## Results

### Study Characteristics

There were 21 studies included in the final analysis, with publication years ranging from 2013 to 2020. Most studies (14/21, 67%) were conducted in the United States. Moreover, of the 21 studies, 2 (10%) were conducted in China, and 2 (10%) other studies were conducted in Sweden. The remaining 14% (3/21) of the studies were conducted in Canada, South Korea, and Spain. The sample size ranged from 17 to 5115 participants, with mean age of 42.44 to 60 years.

The interventions were either developed for general audiences (eg, those who were overweight (BMI >25 kg/m^2^; [26]), for specific audiences with hypertension (eg, patients with poorly controlled hypertension [27], or for patients with diabetes, hypertension, or both [14]. Of the 21 studies, 9 (43%) were randomized control studies [27,36-39,44-47], 4 (19%) were observational studies [26,28,48,49], 6 (29%) were pre-post studies without a control group [25,31,43,50-52], and 2 (10%) were qualitative studies [14,32]. Engagement or self-management behaviors (eg, medication adherence) were not the focus or outcome of longitudinal studies (eg, the study by McGillicuddy et al [28]). In terms of the intervention content, most studies [14,25,27,28,31,32,36-39,43-45,47,48,51] involved medication tracking or medication adherence.

In addition, 24% (5/21) of the studies used a theoretical framework in their interventions. Specifically, some studies [28,38,39] used the self-determination theory. Other theories, including the health belief model and technology acceptance model [43], and the technology-supported apprenticeship model [37] were also applied. No other studies reported any theoretical models. Table 1 presents a summary of the intervention characteristics.

**Table 1:** Intervention characteristics.

Study	Country	Sample size	Participants’ demographic and hypertension characteristics	Duration	Outcomes	Theory
Bengtsson et al [31]	Sweden	50	Mean age 59.5 years; being currently treated for hypertension; mean SBP^a^ 142, mean DBP^b^ 84	56 days or 8 weeks	SBP and DBP, identification of subsets or classes of patients who differed from each other with respect to level of BP^c^ at baseline	No
Chandler et al [38]	United States	54 (IG^d^=26; enhanced standard care=28)	I mean age 44.4 years; enhanced standard group mean age 46.8 years; Hispanic or Latino participants; diagnosed with and prescribed medication(s) for essential hypertension; uncontrolled hypertension	9 months	PO^e^: change in resting SBP from baseline to the 6-mo time point; SO^f^: resting DBP and MA^g^	Self-determination theory
Ciemins et al [49]	United States	IG=131; CG^h^=353	Mean age 60 years; patients with newly diagnosed or persistently uncontrolled BP^h^ (ie≥140/90 mm Hg)	32 weeks	Patients’ compliance with study protocol of taking 3 BPs per week	No
Davidson et al [39]	United States	38 (IG=18; CG=20)	I mean age 47.5 years; 47% (18/38) African Americans and 53% (20/38) Hispanics; uncontrolled hypertension	6 months	Changes in clinic SBP and changes in clinic DBP; changes in SBP control; changes in DBP control; recruitment and retention rates; MA; BP adherence	Self-determination theory
Duan et al [43]	China	143	Aged >18 years, hypertension diagnosis with no other serious complications	2 months	Patient compliance with hypertension self-management	Health belief model and the technology acceptance model
Gong et al [44]	China	480 (IG=225; CG=218)	I mean age 58.2 years; C mean age 59.27 years; patients aged 18 to 79 years diagnosed with primary hypertension	6 months	PO: SBP and DBP changes in patients; change in percentage of participants in the 2 groups with controlled BP. SO: MA	No
Hallberg et al [32]	Sweden	49	Female median age 58 years; male median age 62.5 years; female years with hypertension median 8; male years with hypertension median 6.6	8 weeks	Understanding of the interplay between BP and daily life; motivation to follow treatment	No
Kang and Park [25]	South Korea	38	Mean age 56 years; patients with hypertension who take antihypertensive medications (taking 1 or more antihypertensive drugs)	4 weeks	MA, perceived usefulness, user satisfaction	No
Kaplan et al [48]	United States	5115	Mean age 49 years; mean SBP130 mm Hg; participants who recorded ≥2 BP measurements were included in the study	22 weeks	Use pattern (engagement), efficacy of the app in BP reduction	No
Mao et al [26]	United States	IG=763; CG=73	I mean age 44.78 years; overweight (defined as BMI >25 kg/m^2^; 14.3% (109/763) participants self-reported hypertension	First 4 months of intensive active coaching and 8 months of maintenance coaching	PO: weight loss at 4 months as defined by percent change in total body weight. SO: change in SBP after 4 months of intensive health coaching, as well as the change in number of participants in each hypertensive category from the beginning of enrollment to after 4 months of coaching	No
Márquez Contreras et al [45]	Spain	148 (IG=73; CG=75)	Mean age 57.5 years; patients with mild to moderate arterial hypertension	18 months (with an inclusion period of 6 months and a follow-up of 12 months)	Pharmacological adherence and control of BP in patients with mild to moderate arterial hypertension	No
McGillicuddy et al [28]	United States	IG=8; CG=9	I mean age 42.44 years, C mean age 57.89 years; renal transplant recipients with hypertension with documented medication nonadherence	12 months after the completion of a 3-month randomized control trial	SBP	Self-determination theory
Moore et al [37]	United States	42 (IG=20; CG=22)	Mean age 50.0 years; patients with essential hypertension (average BP≥140/90 and ≤180/120) who were taking 0 or 1 medications	12 weeks	PO: absolute decrease in SBP and DBP and the number of participants who reached the BP goal of ≤130/80 mm Hg. SO: the number of participants who reached the BP goal of ≤140/90 mm Hg, the number of participants who achieved >10 mm Hg decreases in SBP and >5 mm Hg decreases in DBP, the change in medication load, the absolute decrease in weight, the number of patients who lost at least 2.3 kg, hypertension knowledge, satisfaction in care, and the amount of clinician time required in the care	A technology-supported apprenticeship
Morawski et al [27]	United States	IG=209; CG=202	I mean age 51.7 years; C age mean=52.4 years; patients with poorly controlled hypertension	12 weeks	PO: change in self-reported MA and SBP. SO: whether participants had well-controlled BP, defined as 140/90 mm Hg or less	No
Ovbiagele et al [47]	United States	24 (IG=8; CG=16)	Patients with hypertension after stroke	3 months	SBP; emergency department use reduction	No
Patel et al [51]	United States	48	Mean age 53 years; African American 96% (46/48); established essential hypertension; prescribed at least two antihypertensive medications	12-week activation (intervention) phase	PO: MA; SO: MA, level of BP control by clinic measures, pill phone use, patient satisfaction, hypertension medication number and changes during the study period, office visits, emergency room visits, and hospitalization	No
Persell et al [36]	United States	IG=144; CG=153	I mean age 59.6 years; C mean age 58.3 years; adults with uncontrolled hypertension (defined as at least 145 mm Hg systolic or 95 mm Hg diastolic)	6 months	PO: SBP at 6 months. SO: self-reported antihypertensive MA, home monitoring and self-management practices, measures of self-efficacy associated with BP, weight, and health behaviors	No
Petrella et al [46]	Canada	IG=67; CG=60	I mean age 56.7 years; C mean age 59.1 years; participants with at least two metabolic syndrome risk factors	52 weeks including 12 weeks of intervention	PO: SBP and other cardiometabolic risk factors. SO: DBP, waist circumference, lipids (with the exception of high-density lipoprotein cholesterol, which was expected to increase) and markers for blood glucose and inflammation	No
Thies et al [14]	United States	15 out of 22 downloaded the app	Mean age 50 years (22 participants); 27% (6/22) of the patients with diabetes, 18% (4/22) with hypertension, and 55% (12/22) with both	Trial suspended, owing to low enrollment and inconsistent use of the app	The original aim of this study was to evaluate the effectiveness of a commercial mHealth app in improving clinical outcomes for adult patients with uncontrolled diabetes or hypertension, or both. Because of low enrollment and low app use, the project aim was changed to understanding why the trial was unsuccessful	No
Toro-Ramos et al [50]	United States	50	Starters mean age 40.40 years; completers mean age 47.68 years; adults with prehypertension or hypertension	24 weeks	Weight change, BMI change, DBP change, SBP change, hypertension category change	No
Weerahandi et al [52]	United States	17	Mean age 59 years; adults currently taking hypertension medication and had a diagnosis of prehypertension or stage 1 hypertension	13 weeks or 120 days	Engagement and acceptability: the number of blood pressure measurements, weight measurements, and daily steps were logged; the number of coaching phone calls attempted and completed, servings documented in the dietary assessment, and goals set were also assessed. Physiological parameters: BP, heart rate, weight, and steps changes	No

^a^SBP: systolic blood pressure.

^b^DBP: diastolic blood pressure.

^c^BP: blood pressure.

^d^IG: intervention group.

^e^PO: primary outcome.

^f^SO: secondary outcome.

^g^MA: medication adherence.

^h^CG: control group.

### Intervention Strategies

#### Overview

All of the studies used at least two strategies to engage patients. The number of strategies used in the interventions varied from 2 to 6, with a possible maximum of 8. Table 2 provides further details.

**Table 2:** Engagement strategies used in the interventions.

Study	Providing health-related educational information	Reminding or alerting users	Motivational messages or encouragement	Recording and tracking health information	Providing guidance based on information entered by the user	Enabling 2-way communication with clinicians	Providing support through social networks	Supporting behavior change through rewards
Bengtsson et al [31]	No	Yes	Yes	Yes	No	No	No	No
Chandler et al [38]	No	Yes	Yes	Yes	Yes	No	No	No
Ciemins et al [49]	Yes	Unknown	No	Yes	Yes	No	No	No
Davidson et al [39]	No	Yes	Yes	No	No	No	No	No
Duan et al [43]	Yes	Yes	No	Yes	Yes	No	Yes (leaderboard module, version 4)	No
Gong et al [44]	No	Yes	No	Yes	Yes	Yes	No	No
Hallberg et al [32]	No	Yes	Yes	Yes	No	No	No	No
Kang and Park [25]	Yes	Yes	No	Yes	Yes	No	No	No
Kaplan et al [48]	Yes	Yes	Yes	Yes	Yes	No	No	Yes
Mao et al [26]	Yes	Yes	Yes	Yes	Yes	Yes	No	No
Márquez Contreras [45]	No	Yes	No	Yes	No	No	No	No
McGillicuddy et al [28]	No	Yes	No	Yes	Yes	No	No	No
Moore et al [37]	No	No	No	Yes	No	Yes	No	No
Morawski et al [27]	No	Yes	No	Yes	No	No	Yes	No
Ovbiagele et al [47]	No	Yes	Yes	Yes	Yes	No	No	No
Patel et al [51]	Yes	Yes	No	Yes	No	No	No	No
Persell et al [36]	Yes	Yes	Yes	Yes	Yes	Artificial intelligence coaching	No	No
Petrella et al [46]	No	No	No	Yes	Yes	No	No	No
Thies et al [14]	No	No	No	Yes	Yes	Yes	No	No
Toro-Ramos et al [50]	Yes	Yes	Yes	Yes	Yes	Yes	No	No
Weerahandi et al [52]	Yes	No	No	Yes	Yes	Yes	No	No

#### Providing Health-Related Educational Information

A total of 9 studies [25,26,36,43,48-52] provided health-related educational information. The content of education varied, with some studies [36,43,48,49] focusing on hypertension, some studies [25,51] focusing on hypertensive medications, and some studies (eg, the studies by Mao et al [26], Toro-Ramos et al [50], and Weerahandi et al [52]) including dietary approaches to reducing hypertension. However, of the 9 studies, only 1 (11%) [36] specified in the educational materials the reason why self-monitoring is important in BP management and how their control through healthy behavior change is important for lowering the risk of complications.

#### Recording and Tracking Health Information

Some studies [14,25,27,28,31,32,36-38,43,44,47] included features to record both BP and medication adherence or intake. Some other studies included features to record either BP [45,46,48-50,52] or medication intake or adherence [51]. Some studies also recorded other information such as medication side effects (eg, the studies by Bengtsson et al [31] and Hallberg et al [32]), symptom logging (eg, the studies by Bengtsson et al [31], Hallberg et al [32], and Duan et al [43]), and the tracking of diet, heart rate, weight, and steps (eg, the study by Weerahandi et al [52]).

#### Reminding or Alerting Users

Most studies [25,27,28,36,38,39,43-45,47,48,50,51] included reminders for medication intake or BP monitoring, or both. Various reminders focusing on other topics or other types were exercise [44]; weight, diet, exercise, and discomfort [43]; weight, meals and snacks, and physical activity [36]; hospital visit date and input of lifestyle data [25]; clients’ personal goals [26]; appointments [45]; or alerting a *Medfriend* who provides peer support when doses are missed [27]. However, some studies [31,32] included reminders but did not specify the content of reminders.

#### Motivational Messages or Encouragement

There are studies that included motivational messages or encouragement [26,31,32,36,38,39,47,48,50]. For instance, the motivational messages of 1 intervention [38] were designed based on participants’ previous medication adherence levels (ie, nonadherence, partial adherence, and complete adherence) and on their values, beliefs, and long-term or short-term life goals.

#### Providing Support Through Social Networks

A total of 2 studies included the feature to provide support through social networks. In version 4 of 1 app [43], a leaderboard module presenting and comparing the scores generated based on each patients’ self-management behaviors was provided for those users who wanted to enhance their self-management motivation. In another study [27], users were able to designate a *Medfriend*, who was someone else who was granted access to the patient’s medication taking history, received alerts when the patient missed doses, and was able to provide peer support.

#### Supporting Behavior Change Through Rewards

Only 1 intervention [48] included gamification features with a reward system to maximize user interaction. In this app, enthusiastic amination appeared on the screen after each BP recording event [48].

#### Providing Guidance Based on Information Entered by the Users

If cutoffs of BP were exceeded or out-of-range values were observed, patients were contacted [47] or were recommended to take additional BP measurements [28,36,44] or to seek medical attention [44,50]. Health care providers were notified [49] or were called when extreme values were recorded [36] or contacted to follow-up with participants [43,46] and asked to determine the course of action to take with the participant [38] or to make an adjustment to medical regimen as warranted [28,47]. In addition to guidance on out-of-range values, the interventions also helped with solving problems [52]; providing personalized or tailored recommendations or advice [26,36,44]; providing encouragement and suggestions and answering nonpressing questions [14]; providing personalized explanations regarding the stages of hypertension and translation into cardiovascular risk [48]; providing strategies to address behavior change related to calorie reduction, diet improvement, nutrient intake, physical activity increase, and sodium intake reduction [50]; or providing tailored recommendations to users’ questions on lifestyle management (ie, sodium intake, body weight, waist circumference, exercise, alcohol, smoking, and stress) [25].

### Interactivity

Interactivity was analyzed based on providing guidance related to information entered by the users (eg, when abnormal BP values were recorded) and based on whether or not the intervention enabled 2-way regular communication (outside of just specific situations such as when abnormal BP values are observed) between users and a health care professional. A total of 9 studies [28,36,38,43,44,46,47,49,50] included 1-way communication under possible critical conditions. Patients or their health care providers were notified or contacted when out-of-range BP values were reported. Interactivity was limited in the interventions; only 33% (7/21) of the studies provided the possibility of interaction with health care providers or health coaches. In terms of 2-way communication, studies included the possibility of communicating with physicians [14,44], health coaches [26,37,50,52], or an artificial intelligence coach [36]. Users were able to have remote consultations with professional doctors [44] or members of their care team [14]. For instance, a trained coach [50], professionals (licensed nutritionists, physical therapists, and social workers) [26], or master clinicians [37] provided human coaching. For a summary of interactivity, please see Table 3.

**Table 3:** Interactivity, tailoring, and engagement.

Study	Interactivity	Tailoring	Microlevel engagement	Macrolevel engagement
Bengtsson et al [31]	No	Yes	No	No
Chandler et al [38]	Yes	Yes	No	No
Ciemins et al [49]	Yes	No	No	No
Davidson et al [39]	No	Yes	No	No
Duan et al [43]	Yes	Yes	No	No
Gong et al [44]	Yes	Yes	No	No
Hallberg et al [32]	No	Yes	No	No
Kang and Park [25]	No	Yes	No	No
Kaplan et al [48]	No	Yes	Yes	No
Mao et al [26]	Yes	Yes	Yes	No
Márquez Contreras [45]	No	No	No	No
McGillicuddy et al [28]	Yes	Yes	No	No
Moore et al [37]	Yes	Yes	No	No
Morawski et al [27]	No	Yes	No	No
Ovbiagele et al [47]	Yes	Yes	No	No
Patel et al [51]	No	Yes	No	No
Persell et al [36]	Yes	Yes	No	No
Petrella et al [46]	Yes	Yes	No	No
Thies et al [14]	Yes	No	No	No
Toro-Ramos et al [50]	Yes	Yes	Yes	No
Weerahandi et al [52]	Yes	Yes	Yes	No

### Tailoring, Customization, or Personalization

Some level of tailoring was achieved in many studies. All 21 studies included only 1 app version, except 1 (5%) study [43]. Moreover, 4 versions of the app were developed based on users’ disease cognition, self-management experience, and self-management motivation, wherein version 1 had three functional modules (ie, management plan, reminder service, and health checkup), version 2 had four modules (health education was added), version 3 had five modules (health education and health report were added), and version 4 had all six modules (health education, health report, and health report were added) [43].

Some studies [26,46,52] included personalized health goals, such as individualized exercise prescription [46]. The content, information, or features of some interventions was or were customized, based on goals or individual preferences [27,43,50,51], based on antihypertensive medication prescription [31], and based on users’ values and inputs [36].

Some studies [43,44,48] provided personalized feedback or advice in the intervention. For instance, in 1 study, physician’s advice was based on patient’s hypertension self-management experience level [43], and in another study, a personalized explanation of the relationship between stages of hypertension and cardiovascular risk was provided [48]. Interventions with tailored target management recommendations included the studies by Kang and Park [25] and Persell et al [36]. In the study by Moore et al [37], users could make shared decisions about diet, exercise, stress management, and medication with the coach. In some studies, the motivational messages were tailored based on users’ personal preferences [31] and on users’ medication adherence rates, goals, and values and beliefs [38,39]. A total of 2 studies [28,32] included tailored reminders. In some studies [28,47,52], the communication model or channel was customized so that patients were contacted via the preferred mode: SMS text messaging, email, or phone. In 1 study [26], the intervention dose (eg, coaching frequencies) was based on the participants’ needs and availability. For a summary of tailoring, please see Table 3.

### Engagement

In addition to describing the intervention strategies used to engage users, we also explored how engagement has been defined, reflected, and related to biomedical outcomes.

#### How Engagement Has Been Defined

Engagement was defined or operationalized as microlevel interactions with the interventions, but from different dimensions, such as frequency of engagement, time or duration of engagement with the app, and intensity of engagement. A total of 4 studies [26,48,50,52] clearly defined or operationalized engagement (Table 3). Moreover, 1 study [48] defined low engagement (ie, “recording BP for less than 4 weeks”), medium engagement (ie, “recording BP for 4-8 weeks”), and high engagement (ie, “recording BP for longer than 8 weeks”). Another study [26] also defined low (ie, “at the bottom quartile of number of messages and video consults”), medium (ie, “participants in the 25th-75th engagement percentiles”), and high engagement (ie, “top quartile of messages sent per month or number of coaching consults in the 4-month coaching period”). Toro-Ramos et al [50] defined different levels of engagement as starters (ie, “participants who completed at least one lesson per week during the first month, as well as engaged with the health coach (at least once through in app one-on-one messages or through phone calls)”), and completers (ie, “participants who completed at least nine core lessons of 22”). Weerahandi et al [52] defined engagement as “Messages sent to the coach per person, messages sent from the coach per person,” “number of times blood pressure was logged,” “number of times weight was logged,” “number of times steps were logged,” “logged food entries,” and “goals recorded.”

None of the studies we reviewed directly examined or measured users’ subjective experience of engagement, focusing on attention, interest, and affect. Some studies explored users’ subjective experience with a focus on user satisfaction or usability in general using interviews or surveys [38,49], usually conducted at the end of the intervention, which may not objectively capture attention or affect during the intervention, given the broad focus and retrospective nature [53].

#### Behaviors Reflecting Engagement

Although some studies did not clearly state in the articles that they measured engagement with digital behavior change interventions (microlevel) or engagement with behavior change (macrolevel), those behavior-related outcome variables, to some extent, reflected users’ macro- or microlevel of engagement, or both. Two commonly measured behaviors in the outcome variables of the studies were medication adherence and BP self-monitoring. Studies measuring medication adherence used different methods: using technology or devices [38,39,45,51], using self-report or surveys [25,27,36,38,44,51], or using a pharmacy refill rate [51]. Studies [36,38,39,43,46,48,49,52] also used the app or a Bluetooth BP device to measure BP self-monitoring behavior.

In addition to medication adherence and BP self-monitoring, other behaviors were also measured: food or meals logged [36,50,52], messages sent or conversations with the app [14,26,36,52], steps taken [46,52], body weight logging [46,52], frequency of users accessing their weekly BP report [48], number of coaching consults [26], and lessons completed [50].

#### The Relationship Between Engagement and Biomedical Outcomes

Of the 4 studies that clearly defined engagement, 3 (75%) studies [26,48,50] tested and demonstrated the statistical relationship between engagement and biomedical outcomes (ie, weight or BP change), indicating that higher engagement was associated with significantly better biomedical outcomes. However, in 1 study [52], the relationship between levels of engagement and biomedical outcomes was not tested. Among studies that did not explicitly define engagement but included behaviors reflecting engagement, none statistically tested the relationship between the behaviors and biomedical outcomes. However, using patient interviews, 1 study [32] explained the mechanism between engagement and the motivation for macrolevel behavior change: as patients became engaged in graphs or through answering questions and measuring their BP, they were motivated to follow their treatment and understood the interplay between lifestyle and BP. Persell et al [36] did not test engagement but tested the factor crucial to macrolevel engagement or behavior change, self-efficacy [54], or engagement self-efficacy [55], that is, the self-confidence in using the app, controlling BP, knowing when medication changes were needed, and performing nonpharmacologic behaviors to control BP. The study by Persell et al [36] found that self-efficacy in controlling BP was greater in the intervention group.

## Discussion

### Principal Findings

#### Overview

To the authors’ knowledge, this is the first review examining the interactivity, customization, and engagement factors of mHealth interventions for hypertension self-management. This review included 21 studies.

#### Participants

On the basis of the results of the participant inclusion criteria, some studies had very specific criteria, whereas others had very broad inclusion criteria. As participant characteristics (eg, disease type and severity of disease) were quite diverse in some studies, more research is needed to explore the goals, needs, and characteristics of users.

#### Design of Interventions

Engagement or self-management behaviors were not included in the outcomes of the limited longitudinal studies. No studies tested the engagement or self-management behaviors after the mHealth technology was no longer provided. Lack of longitudinal design leads to inability to elucidate behavior change or engagement patterns over time. Without testing macrolevel engagement or self-management behaviors (eg, medication adherence) when interventions or apps are no longer available, it cannot be confirmed that digital behavior change interventions are effective in changing behaviors in the long run.

#### Theoretical Frameworks Applied

Self-determination theory, health belief model, technology acceptance model, and technology-supported apprenticeship models have been applied to a limited number of studies. Some of these theories (eg, self-determination theory) have also been applied to diabetes self-management interventions using mobile apps [56] and applied to mHealth interventions in improving medication adherence among people with hypertension [11]. More interventions should adopt a theoretical framework (eg, behavior change theories) to guide work in this area.

#### Optimal Combination of Engagement Strategies

Many studies have used a combination of features that are likely to engage users. What specific combination of features works best for engagement is yet to be determined, but should consider patients’ characteristics. For instance, patient motivation for self-management may be a factor to consider. Providing patient education content in an app can be a way of engaging patients, especially those who are motivated. However, for those who are not motivated (eg, “motivating participants to read the educational materials remained a challenge,” as indicated by Weerahandi et al [52]), a patient education section may be less beneficial, and other strategies should be considered to motivate patients. Similarly, reminders and motivational messages could be more effective when refined according to patient characteristics and interactions with the app [32,51].

#### Interactivity

Interactivity was limited in digital behavior change interventions, as only 7 interventions provided 2-way communication between users and a health care professional or a coach. Moreover, 9 interventions provided 1-way communication in possible critical conditions; that is, when abnormal BP values were recorded, users or health care professionals were notified. On the basis of the results, the levels of interactivity between users and health care professionals can be characterized into four major categories: no interaction, limited interaction, regular interaction, or focused interaction. Limited interaction includes providing support under possible critical conditions. Regular interaction includes providing the possibility of 2-way communication between users and health care providers (eg, questions and answers and receiving regular feedback and recommendations), along with other strategies or app features. Focused interaction includes providing patient coaching by a clinician or a trained coach, which is the dominant feature, goal of the app, or intervention.

In our review, we found that some apps contained interactivity functions, whereas others did not. Authors did not describe the decision to exclude interactive features; we posit that the decision of including or excluding interactivity might be based on a variety of factors, including patient factors (eg, needs), intervention goals, and health care providers’ availability. For instance, if an app’s aim is medication adherence, interactivity is not a very important feature, whereas if an app’s aim is logging symptoms, especially alarming symptoms, then interactivity (eg, health care providers’ feedback) would be a critical function. In addition, provider-related factors are also worth considering. Studies have demonstrated health care providers’ barriers of using apps to communicate with patients, including time constraints, increased workload, lack of interest, and lack of investment in app development [57].

Some level of potential interactivity should be included in the interventions using mobile apps for hypertension self-management. As 1 article examining patient perspectives indicated, ambiguity and anxiety could be provoked with BP readings, especially when readings are high [7]. Interaction with health care professionals, at least during possible critical points perceived by patients, can be an essential feature to provide professional guidance and ease the concerns and promote engagement with the interventions and the behavior change process [58].

#### Tailoring

The tailoring of interventions varied at different aspects, from tailoring of intervention content (including goals, patient education, advice and feedback from health professionals, reminders, and motivational messages) to tailoring intervention dose and communication mode. Tailoring was carried out in several ways, including consideration of patient characteristics such as goals, preferences, disease characteristics (eg, hypertension stage and medication list), disease self-management experience level, medication adherence rate, values, and beliefs. Although multiple studies included reminders for medication administration, only two of them provided tailored reminders. Medication nonadherence can be due to many factors: medication side effects, cost, forgetfulness, or perceived lack of need to take medications. In addition, personal and cultural values and beliefs (eg, perception of illness, illness knowledge, health literacy, cultural beliefs, self-efficacy, and spiritual and religious beliefs [59]) can impact medication adherence. For instance, higher perceived benefits of herbs and lower perceived benefits of Western medications were predictors of antihypertensive medication nonadherence among Chinese immigrants [60]. A medication reminder feature may therefore work well for those who forget to take medications but may be ineffective for those who do not take medications as prescribed because of costs or potential side effects [61] or those who do not believe in the benefits of Western medications. For those who have high medication adherence rates, reminder features may be redundant or perceived as annoying. To promote medication adherence, intervention content and approach should be tailored according to such personal characteristics. Providing an app with various modules or features and giving the users the ability to select among them may represent an optimal solution for potentially diverse population.

#### Engagement

Engagement was defined or operationalized as a microlevel interaction with the interventions but from different dimensions, such as frequency of engagement, time or duration of engagement with the program, and intensity of engagement. There are a couple of possible reasons why subjective experience of engagement such as attention, affect, and interest were not examined in the studies. Self-report of engagement (subjective measure) at multiple points during the intervention or app use may be disrupting [53] and may create excessive burden for users, especially when they are asked to periodically perform different tasks (eg, logging symptoms and BP and reporting medication adherence) using the app. In addition, objective measures (eg, app use data) can be used to capture attention, interest, or affect during the app use or intervention [55]. For instance, mouse cursor tracking or eye tracking can be used to measure attention [62]. Similarly, digital health intervention use (eg, time spent on education content page and messages sent to the health coach) can be used to measure attention or interest in a nonobstructive way or, to some extent, reflect attention or interest. These could be the reasons why studies used data use to measure engagement but seldom used subjective measure to directly measure attention, affect, or interest.

For all studies that tested this relationship, higher engagement was associated with better biomedical outcomes. However, this result should be interpreted with caution because, first, the result is from 3 applicable studies and, second, although the studies are valuable to the engagement literature, they might have some risk of bias.

Although most studies did not define or focus on engagement per se, their outcome variables reflected engagement with digital behavior change interventions. When reviewing studies of engagement in mHealth, authors should consider seeking out both studies that explicitly mention engagement, as well as studies that do not explicitly define engagement but examine behaviors that reflect engagement.

No studies tested macrolevel engagement directly, but 1 study [36] tested a factor crucial to macrolevel and microlevel engagement or behavior change: self-efficacy or self-confidence (eg, using the app, performing nonpharmacologic behaviors to control BP). In 1 study [7], researchers found that patients with hypertension have various levels of digital competence (defined as “becoming familiar and comfortable with using technology to manage hypertension”) in that some were not interested in using apps for hypertension management and others were digitally competent to use apps. In another study focusing on African American older adults, “participants expressed concerns about not being informed or trained sufficiently to integrate technology for hypertension self-management” [63]. These findings imply that self-efficacy, especially self-efficacy in using technology for disease self-management, can be another important patient factor to be considered when designing interventions [54,55,64].

#### Our Patient-Centered Framework

Overall, the results of this study agreed with those of a prior systematic review [65] of mHealth for self-management of cardiometabolic risk factors, in that some studies are theoretically driven, while direct measurement and evaluation of engagement was limited.

**Figure 2:**
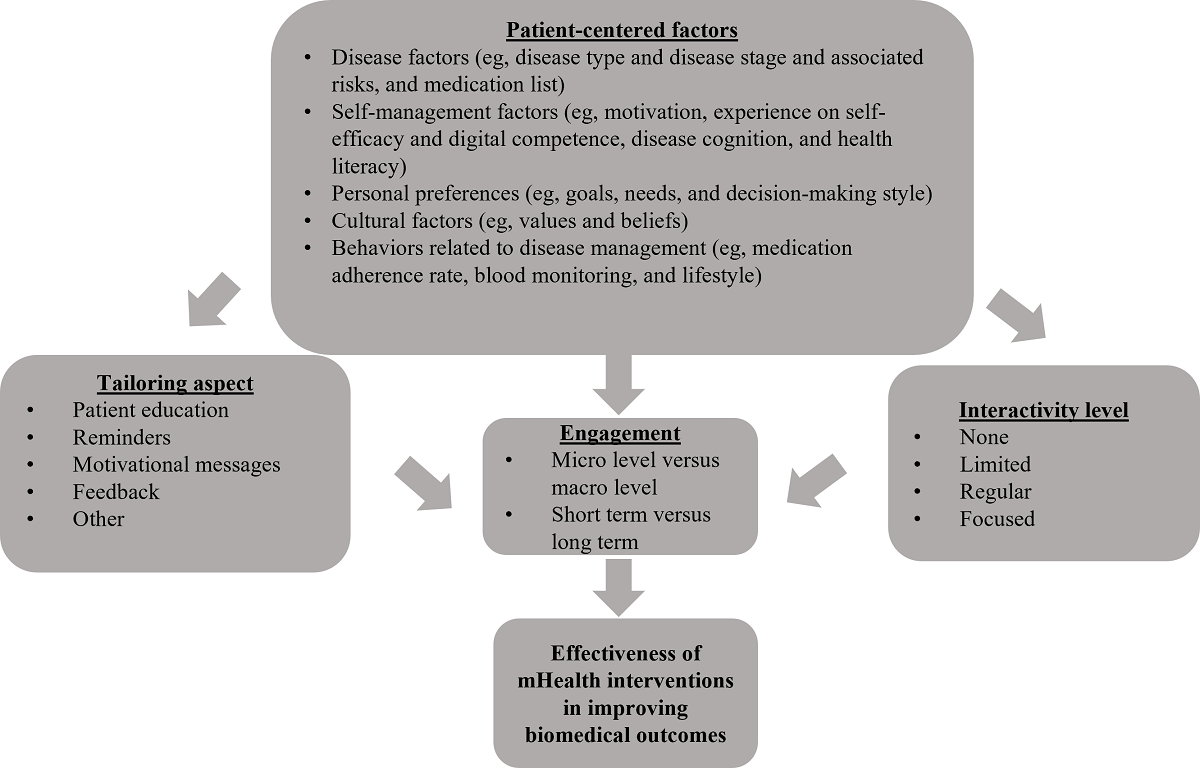
Patient-centered engagement framework for hypertension self-management using mobile health (mHealth) technology.

Considering the definition of engagement [4], the motivational technology model [20], and the results of the review, a framework for the use of mHealth technology for hypertension self-management is proposed (Figure 2). This patient-centered engagement framework emphasizes the important role of patient-centered factors, including but not limited to disease factors, self-management factors, users’ personal preferences, cultural factors, and behaviors related to disease self-management. These factors determine the aspects of an intervention to be tailored and determine the level of interactivity. Patient-centered factors, together with tailoring and interactivity level, determine engagement and subsequently intervention efficacy in improving biomedical outcomes. For instance, Thies et al [14] concluded that their failed intervention was due to lack of attention to patients’ eHealth literacy (defined as “the ability to seek, find, understand, and appraise health information from electronic sources and apply the knowledge gained to addressing or solving a health problem”) [66] and lack of proficiency regarding the chronic disease type. Whereas other engagement frameworks [15] focus on strategies (eg, providing medical information, sending reminders, and tracking health data) to engage patients, our framework highlights categories of patient factors to be considered and how those factors are crucial to customization, interactivity, and engagement.

### Limitations

There are some limitations of this review. Only studies published in English were included in the review, and therefore, there remains potential neglect of important studies published in other languages. Although the authors used a systematic search strategy, other studies meeting the inclusion criteria may have been missed. For instance, those studies not including the key search terms used in the systematic search might be excluded. We were unable to conduct a meta-analysis, owing to the heterogeneity of the studies and outcomes. Further, other factors (eg, navigability) that are also important for engagement and efficacy of interventions were not examined in this review. Given that the proposed patient-centered framework was based on the results of the studies included in the review, it is possible that there are other patient-centered factors (eg, outcome expectation, a significant factor of engagement [55]) that could be important for engagement in the context of hypertension self-management that are not included in the framework.

### Conclusions

Among mHealth app interventions focused on hypertensive management, engagement, interactivity, and tailoring have been implemented in various ways, as demonstrated by the 21 studies included in this review. The authors examined several strategies used to facilitate engagement. The results support the essential roles of engagement in intervention effectiveness and the essential roles of patient factors in tailoring, interactivity, and engagement. A patient-centered engagement framework for hypertension self-management using mHealth technology was proposed, with the intent to help facilitate intervention design and disease self-management using mHealth technology in the future.

## Appendix

Multimedia Appendix 1 Search strategy.

Multimedia Appendix 2 Risk of bias assessment for randomized control trails.

Multimedia Appendix 3 Risk of bias assessment for observational studies.

Multimedia Appendix 4 Risk of bias assessment for pre-post studies without a control group.

Multimedia Appendix 5 Risk of bias assessment for qualitative studies.

## Abbreviations

BP: blood pressure
mHealth: mobile health
NIH: National Institutes of Health
PRISMA: Preferred Reporting Items for Systematic Reviews and Meta-Analyses
