# Supplementary material for: mHealth Interventions for Self-management of Hypertension: Framework and Systematic Review on Engagement, Interactivity, and Tailoring
Source: JMIR Mhealth Uhealth. 2022 Mar 2;10(3):e29415. doi: 10.2196/29415 (PMC8928043; doi:10.2196/29415)
Supplement: Multimedia Appendix 1 [file mhealth_v10i3e29415_app1.docx]

**Multimedia Appendix 1: Search Strategy**

| **Databases** | **Keywords/Search Strategy** | **Justification** | **Results** |
| --- | --- | --- | --- |
| PubMed | (hypertension OR hypertensive OR hypertensives OR blood pressure (title)) AND  (self-management OR self management self-care OR self care OR management OR coaching OR control OR monitor OR adhere OR adherence (title)) AND  (mhealth OR m-health OR mobile OR app OR apps OR application OR applications OR smart phone OR smartphone OR technology (title)) AND  (intervention OR trial OR program OR programme OR experiment OR pilot OR study OR effect OR experience OR experiences (full text)) | The search strategy and the databases chosen were based on the following rationale. 1. We reviewed previously published reviews of similar topic and see what keywords they used; 2. They were based on our research experience: for instance, some interventional studies would not include “intervention” as a word in the title, so searching the keyword in full text would be better than in title. 3. The search strategy were reviewed and approved by experts with clinical and/or mHealth research background. | 115 |
| PsychInfo | (hypertension OR hypertensive OR hypertensives OR blood pressure (title)) AND  (self-management OR self management self-care OR self care OR management OR coaching OR control OR monitor OR adhere OR adherence (title)) AND  (mhealth OR m-health OR mobile OR app OR apps OR application OR applications OR smart phone OR smartphone OR technology (title)) AND  (intervention OR trial OR program OR programme OR experiment OR pilot OR study OR effect OR experience OR experiences (full text)) |  | 10 |
| Embase | (hypertension OR hypertensive OR hypertensives OR blood pressure (title)) AND  (self-management OR self management self-care OR self care OR management OR coaching OR control OR monitor OR adhere OR adherence (title)) AND  (mhealth OR m-health OR mobile OR app OR apps OR application OR applications OR smart phone OR smartphone OR technology (title)) AND  (intervention OR trial OR program OR programme OR experiment OR pilot OR study OR effect OR experience OR experiences (full text)) |  | 161 |
| Mass Media and Communication Complete + CINAHL + Medline + Medline Full text | (hypertension OR hypertensive OR hypertensives OR blood pressure (title)) AND  (self-management OR self management self-care OR self care OR management OR coaching OR control OR monitor OR adhere OR adherence (title)) AND  (mhealth OR m-health OR mobile OR app OR apps OR application OR applications OR smart phone OR smartphone OR technology (title)) AND  (intervention OR trial OR program OR programme OR experiment OR pilot OR study OR effect OR experience OR experiences (full text)) |  | 156 |
