# Supplementary material for: mHealth Interventions for Self-management of Hypertension: Framework and Systematic Review on Engagement, Interactivity, and Tailoring
Source: JMIR Mhealth Uhealth. 2022 Mar 2;10(3):e29415. doi: 10.2196/29415 (PMC8928043; doi:10.2196/29415)
Supplement: Multimedia Appendix 2 [file mhealth_v10i3e29415_app2.docx]

**Multimedia Appendix 2: Risk of Bias Assessment for Randomized Control Trials**

| **Randomized Control Trial** | **1. Random Sequence Generation** | **2. Allocation Concealment** | **3. Blinding of Participants and Personnel** | **4. Blinding of Outcome Assessment** | **5. Incomplete Outcome Data** | **6. Selective Reporting** | **7. Other Bias** |
| --- | --- | --- | --- | --- | --- | --- | --- |
| Chandler et al., 2019 | ? | ? | x (unable to blind) | ? | √ | √ | x |
| Davidson et al., 2015 | ? | ? | x (unable to blind) | ? | x | √ | x |
| Gong et al., 2020 | √ | ? | x (unable to blind) | ? | √ | √ | √ |
| Marquez Contreras et al., 2018 | √ | √ | x (unable to blind) | ? | √ | √ | √ |
| Moore et al., 2014 | √ | √ | x (unable to blind) | ? | √ | √ | x |
| Morawski et al., 2018 | √ | x | x (unable to blind) | √ | √ | √ | x |
| Persell et al., 2020 | √ | √ | x (unable to blind) | √ | √ | √ | ? |
| Petrella et al., 2014 | x | x | x (unable to blind) | x | x | √ | √ |
| Ovbiagele et al., 2015 | ? | ? | x (unable to blind) | ? | ? | √ | x |

√: low risk of bias, x: High risk of bias,?: unclear risk of bias
