# Supplementary material for: mHealth Interventions for Self-management of Hypertension: Framework and Systematic Review on Engagement, Interactivity, and Tailoring
Source: JMIR Mhealth Uhealth. 2022 Mar 2;10(3):e29415. doi: 10.2196/29415 (PMC8928043; doi:10.2196/29415)
Supplement: Multimedia Appendix 4 [file mhealth_v10i3e29415_app4.docx]

**Multimedia Appendix 4: Risk of Bias Assessment for Pre-Post Studies With No Control**

| **Pre-Post studies With No Control** | **Toro-Ramos et al., 2017** | **Patel et al., 2013** | **Bengtsson et al., 2016** | **Duan et al., 2020 (Quantitative Part)** | **Kang et al., 2016** | **Weerahandi et al., 2020** |
| --- | --- | --- | --- | --- | --- | --- |
| 1. Was the study question or objective clearly stated? | √ | √ | √ | √ | √ | √ |
| 2. Were eligibility/selection criteria for the study population prespecified and clearly described? | √ | √ | √ | √ | √ | √ |
| 3. Were the participants in the study representative of those who would be eligible for the test/service/intervention in the general or clinical population of interest? | x | x | √ | x | x | x |
| 4. Were all eligible participants that met the prespecified entry criteria enrolled? | NR | x | x | NR | NR | NR |
| 5. Was the sample size sufficiently large to provide confidence in the findings? | √ | √ | √ | NR | x | x |
| 6. Was the test/service/intervention clearly described and delivered consistently across the study population? | √ | √ | √ | √ | √ | √ |
| 7. Were the outcome measures prespecified, clearly defined, valid, reliable, and assessed consistently across all study participants? | CD | CD | CD | CD | CD | CD |
| 8. Were the people assessing the outcomes blinded to the participants' exposures/interventions? | NR | NR | NR | NR | NR | NR |
| 9. Was the loss to follow-up after baseline 20% or less? Were those lost to follow-up accounted for in the analysis? | √ | √ | √ | NR | x | √ |
| 10. Did the statistical methods examine changes in outcome measures from before to after the intervention? Were statistical tests done that provided p values for the pre-to-post changes? | √ | √ | √ | NR | √ | √ |
| 11. Were outcome measures of interest taken multiple times before the intervention and multiple times after the intervention (i.e., did they use an interrupted time-series  design)? | x | √ | √ | √ | x | x |
| 12. If the intervention was conducted at a group level (e.g., a whole hospital, a community, etc.) did the statistical analysis take into account the use of individual-level data to determine effects at the group level? | NA | NA | NA | NA | NA | NA |

√: Yes; x: No; NR, not reported; NA, not applicable; CD, cannot determine
