# Supplementary material for: mHealth Interventions for Self-management of Hypertension: Framework and Systematic Review on Engagement, Interactivity, and Tailoring
Source: JMIR Mhealth Uhealth. 2022 Mar 2;10(3):e29415. doi: 10.2196/29415 (PMC8928043; doi:10.2196/29415)
Supplement: Multimedia Appendix 5 [file mhealth_v10i3e29415_app5.docx]

**Multimedia Appendix 5: Risk of Bias Assessment for Qualitative Studies**

| **Qualitative Study** | **Hallberg et al., 2016** | **Thies et al., 2017** | **Duan et al., 2020 (Qualitative part)** |
| --- | --- | --- | --- |
| 1. Was there a clear statement of the aims of the research? | √ | √ | √ |
| 2. Is a qualitative methodology appropriate? (broad sense) | √ | √ | √ |
| 3. Was the research design appropriate to address the aims of the research? | √ | √ | √ |
| 4. Was the recruitment strategy appropriate to the aims of the research? | √ | √ | NR |
| 5. Was the data collected in a way that addressed the research issue? | √ | √ | √ |
| 6. Has the relationship between researcher and participants been adequately considered? | NR | NR | NR |
| 7. Have ethical issues been taken into consideration? | √ | √ | √ |
| 8. Was the data analysis sufficiently rigorous? | √ | √ | x |
| 9. Is there a clear statement of findings? | √ | √ | √ |
| 10. How valuable is the research? | Valuable | Somewhat valuable | Somewhat valuable |

√: Yes; x: No; NR, not reported
